# The Capability Approach and Upstream Causes—A Discussion of Johnson’s Ethical Evaluation Framework for Coercive Antimicrobial Stewardship Policies

**DOI:** 10.1093/phe/phaf013

**Published:** 2025-09-19

**Authors:** Simon H Bangma, Karlijn M L Rensink

**Affiliations:** Philosophy, Bioethics and Health, VU Amsterdam University, Amsterdam, The Netherlands; Philosophy, Bioethics and Health, VU Amsterdam University, Amsterdam, The Netherlands

## Abstract

The rise of antimicrobial resistance (AMR) poses a major global health threat. One of the ways to address this issue is through coercive enforcement of antimicrobial stewardship (AMS) policies. The use of such (state) coercion requires ethical justification. Therefore, Dr. Tess Johnson has recently developed a framework to evaluate the possible ethical justifications for these coercive AMS policies. In this discussion article, we aim to contribute to further developing Johnson’s ethical evaluation framework by providing two additional arguments. First, we remark how the high burden of ethical justification is premised on the assumption that coercion restricts freedom. We argue that this only applies to narrow conceptions of freedom, while not necessarily to broader conceptions, such as the capability approach. Second, we advocate for attentiveness to upstream causes of AMR. We argue that ethically justified coercive AMS policy is conditional upon giving sufficient consideration to solving these upstream causes.

## Introduction

Antimicrobial resistance (AMR) is a major global health threat to humanity (Naghavi *et al.*, 2024). Therefore, appropriate use of existing antimicrobials, known as antimicrobial stewardship (AMS), is essential to mitigate the problem of AMR (World Health Organization, 2023). Adequate AMS policies might require (state) coercion, because exclusively non-coercive policies are, arguably, unable to achieve socially desirable outcomes. Johnson (2024) developed an ethical framework to evaluate ethical justifications that policy-makers can use for coercive AMS policies. We agree with this comprehensive framework and especially appreciate its evaluative component. However, we argue that two important arguments can be of added value in the development of this ethical framework. First, the moral wrongness of coercion—and consequently the need for ethical justification—is premised on the assumption that it restricts freedom. We argue this only applies to narrow conceptions of freedom, while not necessarily to others, such as the capability approach. Second, we emphasise the moral significance of so-called ‘upstream causes’ and propose an additional overarching precondition for coercive AMS policy; only if sufficient consideration has been delegated to solve upstream causes of AMR, coercive AMS policy is ethically justifiable.

## Coercion and Freedom

An important charge many public health policymakers will face when implementing coercive policy is that such measures involve an undue infringement on (individual) freedom. Especially in liberal democratic traditions, as Johnson notes, coercion is seen as ethically problematic due to its supposed restriction on freedom (Johnson, 2024, pp. 11–12). The coercer, usually the state, coerces the coercee by deliberately interfering within an area in which the coercee could otherwise have acted (Berlin, 2002 [1958]). If it is assumed that coercion *always* restricts freedom, then one is implicitly committed to the narrow, non-interference conception of freedom. By hypothesis, coercion involves deliberate interference; therefore, it must always restrict freedom. Accordingly, if we value freedom—as we do in liberal democracies—one must conclude that coercion is *pro tanto* wrong.

However, Johnson rightfully contends that freedom is not the only value at stake; coercive policies can be justified when other values that we find important, such as efficiency, fairness or justice, sufficiently outweigh the moral wrongness of the assumed restriction of freedom (Johnson, 2024, p. 13). We will refer to this as the ‘outweighing strategy’ (Schmidt, 2022). It holds that coercion may restrict freedom, but that freedom is not absolute and can be outweighed by other values or moral principles. In other words, it satisfies the justificatory condition of proportionality (Childress et al., 2002, p. 173). The outweighing strategy is widely accepted in public health, as even those highly valuing individual freedom (e.g. libertarians, classical liberals) acknowledge that pressing public health claims can override claims to freedom. Notably, by committing to the outweighing strategy, one does not challenge the assumption that coercion restricts freedom.

However, as noted, this assumption is contingent upon using a narrow, non-interference conception of freedom. We argue that the assumption can be refined by a more nuanced, broader conception of what freedom entails. We show this by considering an alternative conception of freedom: the capability approach. This approach argues that ‘real’ or ‘genuine’ freedom consists of the *ability* to reach valuable states of well-being (i.e. ‘functionings’) (Robeyns and Byskov, 2023). It criticises the traditional, classical liberal, non-interference conception of freedom ‘for insufficiently attending to the material and institutional prerequisites of genuine freedom’ and ‘[to] fetishize freedom as an all-purpose social good’ (Nussbaum, 2006, p. 216). According to Nussbaum (2000), who developed a dominant application of the capability approach, every human is entitled to ten universal capabilities necessary for a flourishing life worthy of human dignity.^1^ Nussbaum’s list illustrates a central aspect of any capability theory: the distinction between valuable and non-valuable freedoms (Robeyns, 2017, pp. 41–45). Valuable freedoms, such as those on Nussbaum’s list, might be worthy of institutional protection, while others are not. Therefore, coercive policies are perfectly justified when they protect valuable freedoms or restrict non-valuable, harmful freedoms.^2^ Thus, if one follows a capabilitarian conception of freedom, then one must be open to the possibility that there are freedom-based reasons supporting coercion. This would result in a decreased burden of ethical justification.

To illustrate, consider a typical, coercive AMS policy that restricts the use of certain human-critical antibiotics in livestock, as discussed by Johnson (2024, p.18). Such a policy might reduce the efficacy of industrial meat production but also AMR-related mortality and morbidity, especially of those vulnerable to resistant pathogens. A policymaker could argue that the capabilities related to efficient meat production (e.g. ‘buying cheaper meat’) are subordinated to the capability of ‘enjoying sufficient health’.^3^

The point here is not that a policymaker can cherry-pick a conception of freedom that happens to accommodate the desired policy; the point is that it should not be assumed *beforehand* that coercion restricts freedom. We remain agnostic about the preferred conception of freedom. However, we want to emphasise how the conception of freedom one follows influences the moral wrongness of coercion—and consequently the need for ethical justification. Thus, an ethical framework should not automatically be premised on the assumption that coercion is always morally wrong.

## Attentiveness to Upstream Causes

Second, we argue that an ethical framework ought to emphasise the moral significance of policies affecting so-called ‘upstream’ causes of AMR. The upstream–downstream metaphor, credited to Zola (1972) and described by McKinlay (1979), addresses the importance of the underlying causes of public health issues; we should prevent people from being pushed into the ‘river of illness’ instead of merely trying to save them downstream. In this context, it is important to highlight that AMR can not only be addressed through (coercive) AMS policies but also by developing new antibiotics or alternative therapies (Johnson, 2024, p. 11). Assuming one wants to avoid using coercion in public health, all else being equal, one also ought to be committed to policies that prevent situations that necessitate coercion being used (Smith, 2024).

Current AMR policy efforts do not seem to reflect such a commitment. Consider, for example, the opening statement of the reasoning that was used to justify the earlier-mentioned restriction of antibiotics being used in livestock: ‘Given the limited innovation in developing new antimicrobials, (…)’ (Johnson, 2024, p. 18). The policymaker *a priori* rules out policies stimulating the development of new antimicrobials by situating ‘limited innovation’ as an irreversible fact (i.e. ‘given’). The opposite seems to be true; in fact, the central problem of the empty ‘drug development pipeline’ is not scientific limitations but a lack of economic research and development (R&D) investments (Klug *et al.*, 2021). This is an upstream determinant that can be affected by adequate public health policy.

This is only one example of how underlying ‘systems of rules and institutions’ (which we define as ‘upstream causes’) are left unaddressed (Chater and Loewenstein, 2023, p. 2). Hence, some scholars argue we should ‘refocus’ to the upstream, systematic causes of public health problems that currently receive insufficient attention (McKinlay, 1979; Chater and Loewenstein, 2023). An ethical framework that does *not* address these upstream causes risks preserving this lack of attention.

Hence, the policymaker ought to be sufficiently attentive to the *potential* of upstream policies, both for avoiding coercion and for being ethically preferable over downstream policies. To illustrate, a policy that incentivises (or mandates) pharmaceutical companies to appropriate R&D investments in antimicrobials can be more effective at solving AMR (cf. Johnson’s ‘Effectiveness’ justification) and live up to corporate social responsibility requirements (cf. Johnson’s ‘Responsibility-Tracking’ justification). *A priori* negligence of such a policy is not innocuous but ethically objectionable. Hence, an ethical framework ought to include a ‘duty of consideration’ regarding upstream causes as an overarching precondition for the ethical justifiability of coercive AMS policy.^4^

## Conclusions

Public health policies that adequately mitigate the undesirable consequences of AMR presumably involve some form of state coercion. In this article, we aimed to broaden the scope of a possible ethical framework for these coercive policies. First, we articulated the possibility of freedom-based reasons supporting coercive policy, illustrated by the capabilitarian conception of freedom. Second, we emphasised the importance of sufficient attentiveness to upstream causes of AMR, exemplified by the empty economic pipeline. We hope these considerations contribute to the development of a comprehensive ethical framework, ultimately guiding the policymaker to ethically appropriate AMS policy.

## Footnotes

1 These are life, bodily health, bodily integrity, senses, imagination and thought, emotions, practical reason, affiliation, other species, play, and control over one’s environment (Robeyns and Byskov, 2023).

2 Other theories of justice, for which we do not have space in this paper, also differentiate between the importance and value of different liberties. Consider, for example, John Rawls’s distinction between basic liberties and non-basic liberties (Wenar, 2021).

3 For another application of the capability approach in a similar context, see Bernstein and Navin (2023). For another example of freedom-based reasoning in favour of coercive policy, see Schmidt (2022).

4 We recognise that our argument faces both practical and conceptual challenges. Practically, upstream policies may be harder to implement or fall outside the authority of local policymakers. Conceptually, the distinction between ‘upstream’ and ‘downstream’ policies can be ambiguous, especially when policies are accessed across time, rather than at one point in time. Also, causal interference is often a complex, interrelated ‘web’ rather than a linear, vertical upstream–downstream interaction. To avoid these challenges, we only propose a duty of consideration. Further research could investigate whether additional, more stringent duties should be included in the ethical framework.
